# Machine Translation of Public Health Materials From English to Chinese: A Feasibility Study

**DOI:** 10.2196/publichealth.4779

**Published:** 2015-11-17

**Authors:** Anne M Turner, Kristin N Dew, Loma Desai, Nathalie Martin, Katrin Kirchhoff

**Affiliations:** ^1^ Northwest Center for Public Health Practice Department of Health Services University of Washington Seattle, WA United States; ^2^ Northwest Center for Public Health Practice Human Centered Design & Engineering University of Washington Seattle, WA United States; ^3^ Northwest Center for Public Health Practice Information School University of Washington Seattle, WA United States; ^4^ Northwest Center for Public Health Practice Department of Biomedical Informatics and Medical Education University of Washington Seattle, WA United States; ^5^ Speech, Signal and Language Interpretation (SSLI) Lab Department of Electrical Engineering University of Washington Seattle, WA United States

**Keywords:** public health informatics, public health, natural language processing, machine translation, Chinese language, health promotion, public health departments, consumer health, limited English proficiency, health literacy

## Abstract

**Background:**

Chinese is the second most common language spoken by limited English proficiency individuals in the United States, yet there are few public health materials available in Chinese. Previous studies have indicated that use of machine translation plus postediting by bilingual translators generated quality translations in a lower time and at a lower cost than human translations.

**Objective:**

The purpose of this study was to investigate the feasibility of using machine translation (MT) tools (eg, Google Translate) followed by human postediting (PE) to produce quality Chinese translations of public health materials.

**Methods:**

From state and national public health websites, we collected 60 health promotion documents that had been translated from English to Chinese through human translation. The English version of the documents were then translated to Chinese using Google Translate. The MTs were analyzed for translation errors. A subset of the MT documents was postedited by native Chinese speakers with health backgrounds. Postediting time was measured. Postedited versions were then blindly compared against human translations by bilingual native Chinese quality raters.

**Results:**

The most common machine translation errors were errors of word sense (40%) and word order (22%). Posteditors corrected the MTs at a rate of approximately 41 characters per minute. Raters, blinded to the source of translation, consistently selected the human translation over the MT+PE. Initial investigation to determine the reasons for the lower quality of MT+PE indicate that poor MT quality, lack of posteditor expertise, and insufficient posteditor instructions can be barriers to producing quality Chinese translations.

**Conclusions:**

Our results revealed problems with using MT tools plus human postediting for translating public health materials from English to Chinese. Additional work is needed to improve MT and to carefully design postediting processes before the MT+PE approach can be used routinely in public health practice for a variety of language pairs.

## Introduction

A key role of public health departments is to inform and educate the public on issues of public health importance. Health departments produce health promotion materials on a range of topics, such as environmental health, communicable diseases, immunizations, and maternal-child health, and the Internet has become a key mechanism by which they distribute and disseminate this information. Although federal and state regulations require that health materials be made available in the languages of patients, due to the time and costs required to manually produce quality translations, very few of these materials are available in languages other than English [1]. Therefore, individuals with limited English proficiency (LEP) have limited access to this health information. This is of particular significance given that LEP status is associated with poor health literacy and negative health consequences, including documented health disparities such as poorer health outcomes and poorer access to health care and preventive services compared to English-speaking minorities [2-4].

Machine translation (MT)—the automatic translation of text from one human language into another by a computer program—has been an area of study within natural language processing for several decades. State-of-the-art MT tools use a statistical machine translation (SMT) framework. This approach uses large amounts of parallel text for the desired language pair to train SMT models. During testing, an SMT engine then produces the most likely translation under the statistical model. While MT tools have improved greatly over the last 5 years, and MT is now routinely used by many language service providers, the quality of raw MT output generally falls short of human-generated translations (HT).

In order to produce quality translations, MT errors need to be corrected by human readers who have domain expertise and are fluent in the source and target languages. This correction, called postediting (PE), can range from light to heavy editing. It has been shown that MT+PE increases productivity (ie, it can be completed more quickly than producing an entirely new HT) both for translators and for lay users [5]. However, compared with translating, postediting is a cognitively different process, and postediting results are strongly dependent on posteditor skill, attitudes towards machine translation, difficulty of the source document, and quality of the initial machine translation output [5,6].

Our previous research indicates that freely available MT tools, such as Google Translate and Microsoft Translator, can be used in conjunction with human PE to produce quality translations efficiently and at low cost [7,8]. We compared the time and cost of HT versus MT+PE for Spanish public health documents, using health professionals as posteditors [7]. Posteditors corrected 25 machine-translated public health documents. Pairs of HT and MT+PE were blindly presented to 2 bilingual public health professionals, who were asked to rate which of the translations they preferred. In this blinded rating, the HT and MT+PE were found to be overall equivalent (33% HT preferred, 33% MT+PE preferred, 33% both translations considered equivalent).

These previous studies were conducted on a single language pair of English-Spanish. SMT generally works best when the source and target languages have similar sentence structures, as in the case of English-Spanish. In order to assess the broader usefulness of MT technology in public health departments, it is necessary to determine whether these results generalize to a wider set of language pairs, specifically those pairs with very divergent linguistic structures. One such pair, English-Chinese, is of particular interest since Chinese is the second most common language spoken by LEP individuals in the United States, representing 6.1% of the LEP population [9].

We conducted postediting experiments, similar to those conducted for the English-Spanish pair, in order to determine the feasibility (accuracy and efficiency) of using MT+PE for translating public health documents from English to Traditional Chinese. We investigated the types of MT errors occurring in Chinese, the PE time needed to correct them, and the quality of MT+PE compared to HTs, as rated by raters fluent in both English and Traditional Chinese. In this paper, we discuss the results of these investigations and compare them to our previous experiences with the English-Spanish pair. This work contributes to our understanding of the challenges involved in applying the MT+PE approach in a public health setting.

## Methods

### Initial Steps

**Figure 1:**
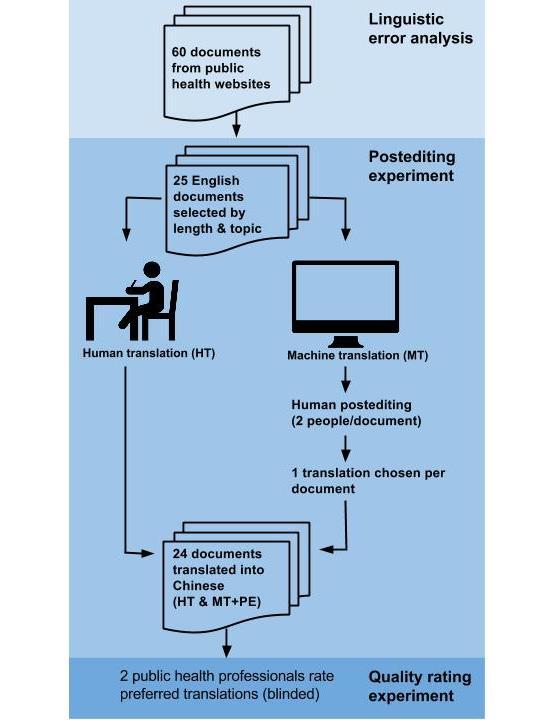
Study Design Overview.

We collected 60 health promotion documents from different public health agencies in the United States that had been translated manually (HT) from English to Chinese. Translations were created using the Traditional Chinese character set, as opposed to Simplified Chinese, because this is the form known to most Chinese LEP individuals in the Pacific Northwest region. We identified the types of linguistic errors present in MT from English to Chinese and then conducted the postediting of the translated materials with participants fluent in both languages. Next, we had bilingual public health professionals and laypersons rate the quality of the human versus the MT plus postedited documents. A diagram of the study design is shown in Figure 1. A more detailed description of the specific methods for the linguistic error analysis, postediting and rating studies, and follow-up evaluation is provided below.

### Linguistic Error Analysis

We collected 60 health promotion documents available in English and Chinese (Traditional) from public health websites in the United States. Websites included those of the Centers for Disease Control and Prevention, New York City Department of Health and Public Health, Minnesota Department of Health, Washington State Department of Health, Department of Public Health – Los Angeles County, and Public Health – Seattle & King County. All Chinese versions of these documents had been translated manually (HT) by health department translators or professional translation vendors. The English versions of the documents were then translated into Traditional Chinese using Google Translate. We developed a categorization scheme for MT errors, and all MTs were annotated based on this scheme by a native Chinese speaker with formal training in linguistics. Subsequently, aggregate error statistics were computed to gain insights into the most frequent error categories: word sense, word order, missing word, superfluous word, orthography/punctuation, particle error, untranslated word, pragmatic error, and other grammar error.

### Postediting Experiments

For the postediting studies, we selected 25 of the 60 health documents that had been machine translated from English to Chinese using Google Translate. To ensure a wide representation of topics, we selected the documents based on the length of the English version (340-914 words) and topic area. From the memberships of local Chinese cultural organizations, 6 Chinese translators were recruited for postediting and screened for language ability and health experience. Posteditors, all native Chinese speakers, were fluent in oral and written Traditional Chinese and English, had varying levels of translation experience, and had prior experience in a health-related field (Table 1).

The 25 machine-translated documents were each corrected by at least 2 posteditors in order to permit consistency checks across posteditors and computation of average time, adequacy, and fluency ratings per document. Posteditors used a proprietary MT and postediting tool built for the purpose of this study, as described previously [7]. Each posteditor corrected between four and 21 documents representing common types of public health materials, including informational webpages, agency letters, fact sheets, and brochures. Posteditors were allowed to choose their preferred character input method. One posteditor used a pinyin keyboard called Q9, while the rest used the standard Windows OS pinyin input. The postediting tool displays three versions of the text from left to right in one window: the original English text, the MT, and the editable MT, respectively. When a posteditor clicks the editable MT field to begin editing, a timer starts. The tool saves the total editing time (minus pauses), keystrokes, and a copy of the postedited machine translation. Time and keystroke data were collected for all postedited documents. Due to a posteditor saving error, only 24 of the 25 postedited documents were put out in a readable format and therefore available for rating.

Posteditors were given written and verbal instructions to “perform all corrections necessary to ensure that the text (1) is consistent with the grammar rules of Chinese, (2) adequately represents the meaning of the English text, (3) is culturally appropriate (ie, not unintentionally funny or offensive), and (4) preserves the linguistic style of the source document.” Posteditors were asked not to alter a correct, appropriate translation simply because it may not correspond to their first choice of translation. In short, they were instructed to correct only as much as necessary and to not rewrite the text. These were the same instructions used in the previous Spanish study.

After completing postediting, participants were asked to fill out a questionnaire to rate the adequacy and fluency of each MT+PE on a scale of 1-5. These rating scales are common in human evaluations of machine translation quality [10]. An adequacy of 1 indicated that none of the original meaning of the English source text was retained in the MT, while an adequacy of 5 indicated that all of the meaning was retained. A fluency rating of 1 indicated that the MT was incomprehensible, while a rating of 5 indicated flawless Chinese. The questionnaire also asked participants to describe the common translation errors they found, identify which errors were most difficult to correct, and explain which errors took the longest time to correct.

### Quality Rating

Two public health professionals, blinded to the method of translation, compared the quality of the postedited documents to the quality of the HT documents from the health department websites. The quality raters were asked to rate the MT+PE against HT versions. One rater was a professional public health translator and a Department of Social and Health Services Certified Medical Interpreter at a local clinic; the other was a health researcher (Table 1). They were presented with 20 sets of documents selected from the 24 available, with each set containing an original English text, an HT version of that text, and an MT+PE version of the text. Even though one rater participated in the initial postediting study as well, she did not rate documents that she had encountered while postediting. The documents were not labeled as human- or machine-translated, and the order in which they were presented in each set was randomized. Using a questionnaire, we asked the quality raters to read each set carefully, indicate which of the translated versions they preferred, and describe why they chose that version, based on five dimensions: grammar, adequacy, word choice, cultural appropriateness, and reading level.

**Table 1:** Initial postediting and quality rating participants, health, and translation experience.

Participant number	Role	Health background	Translation experience
P1	Posteditor	Pharmacy student	Limited—translating at health fairs
P2	Posteditor	Social work for Chinese population, including health care support	Teaching English as a second language & translating research
P3	Posteditor and quality rater	Public health researcher	10 years of various translation experience
P4	Posteditor	Social work for Chinese population, including health care support	Translating agency and government publications for distribution to clients
P5	Posteditor	Public health student	None
P6	Quality rater (posteditor for follow-up evaluation only)	Public health translator	DSHS Certified Medical Interpreter

### Follow-Up Evaluation

After analyzing the results of the quality rating study, we performed follow-up evaluations of the effects of posteditor expertise, engagement, and instructions on the quality of postedited translations. To assess whether posteditors’ public health and translation expertise negatively impacted the quality rating outcome, we asked P6, a highly trained and experienced health translator, to postedit four documents. We then repeated the quality rating procedure with those documents, asking five native Chinese speakers to review them.

To test posteditor engagement and whether the instructions to edit only as necessary were problematic, we asked 3 posteditors (P2, P4, and P5) to return to edit a total of 10 more documents, this time with instructions to make as many corrections as needed to ensure the quality of the translation. We again repeated the quality rating procedure with one native Chinese speaker who has public health experience to see whether posteditors given the revised instructions would produce text equivalent to the HTs.

## Results

### Linguistic Error Analysis

Results from the linguistic error analysis are summarized in Table 2. The left-hand column shows the error type; the right-hand column shows the corresponding frequency of the error type, computed as the percentage of all errors annotated in the total set of 60 documents. For example, word sense errors (errors where the word meaning was translated incorrectly) constituted 40% of all annotated errors. The next most common error types involved word order (22%) and missing words (16%).

**Table 2:** Error categories and their distributions.

Error categories	Frequency (%)
Word sense	40
Word order	22
Missing word	16
Superfluous word	14
Other grammar error	3
Orthography/punctuation	3
Particle error	1
Untranslated word	0.03
Pragmatic error	0.01

### Postediting Experiments

The proprietary postediting tool recorded the time taken to postedit each machine-translated document. We analyzed the time taken, by document and by posteditor, and examined posteditors’ quality ratings of the initial MT output. A list with descriptions of the source documents is provided in Multimedia Appendix 1.

To determine and analyze the amount of time required for postediting, we calculated the number of characters per minute (CPM) for each document and then computed means and standard deviations (SDs) in CPM for each document, using posteditors’ recorded times. In addition, we computed means and SDs in CPM for each posteditor (Table 3). This helped us gain insights into potential correlations between postediting time and document topic, length, etc, as well as differences between posteditors (though not all posteditors edited the same number of documents).

The mean CPM per document varied greatly, from 18.5-79.6 CPM (SD 0.03-38.7). The total mean CPM across all documents was 37.8 (SD 10.2). Thus, on average a posteditor corrected approximately 38 CPM, with a variation of around 10 CPMs. The results did not indicate a linear relationship between document length and average postediting time. We also found no relationship between the document type and the average CPM.

On average, the posteditors rated the adequacy of the translations at 3.32 (SD 0.90), suggesting that much of the original meaning of the source text was preserved in the MT. Average fluency rating was 3.0 (SD 0.84), which corresponds to a grammar quality level of non-native Chinese. The average adequacy and fluency ratings bore no relationship to the document type or length, but varied greatly by individual posteditor. Interestingly, the posteditors who had more experience with translation and health rated the adequacy and fluency lower than did their less experienced counterparts (Table 3).

To investigate the variation in postediting speed for individuals, we calculated the average CPM for each posteditor. As shown in Table 3, the average CPM was 37.4 and the average SD for CPM per document was 15.7. We also found large individual differences in speed among posteditors [11,12]. Posteditors also varied widely in their adequacy and fluency ratings, with a trend indicating an inverse relationship between public health translation experience and ratings; the more experienced posteditors in terms of translation and public health expertise tended to rate the documents they postedited lower than those with less experience (Tables 1 and 3).

Errors described by posteditors as difficult to correct, or annoying, included word sense errors and word order errors. Some examples of the errors noted by posteditors are provided in Table 4.

**Table 3:** Postediting time, adequacy, and fluency ratings by posteditor.

Posteditor	Docs postedited, n	CPM, mean (SD)	Avg. adequacy	Avg. fluency
P1	9	34.2 (7.3)	4	3.2
P2	21	35.4 (16.2)	N/A	N/A
P3	4	25.8 (10.2)	3	2.5
P4	4	54.3 (40.5)	3.25	3.25
P5	11	54.0 (16.0)	3.875	3.75
P6	4	20.6 (3.7)	1.75	1.625

**Table 4:** Posteditor examples of top three error categories.

Error category	Quotes/examples
Word sense	“The literal meaning changes when translated into Chinese (eg, lost power/electricity is translated as lost ‘energy’)”
Word order	“‘...when...can’t...’ type of sentence doesn’t have same structure in Chinese. The order of the words change in Chinese and English in many situations”
Missing word	“Whenever there is the word ‘person’ we should mention ‘this’ or ‘that’ person, otherwise it is not clear who are we talking about in the sentence.”

### Quality Rating

Unlike our previous experience with English to Spanish translations, in a blind comparison of HT and MT+PE, the quality raters selected the HT document as the preferred version for all 20 documents. Reasons given for the preference were better word order, a more professional reading level, smoother flow, more accurate translated word use, preserved meaning, and cultural appropriateness of the original English document. The reasons the rater gave for rejecting the MT+PE documents were that they did not meet the reading level of the general public, some of the sentences lost the intended meaning, the same words were not translated consistently, awkward word order, and occasionally wrong word translations and awkward word flow.

### Follow-Up Evaluation

In theory, if posteditors have sufficient training, experience, and resources to perform quality postediting, MT+PE documents should be equivalent to HT documents. The feasibility of utilizing MT+PE has been repeatedly demonstrated in various previous studies for a variety of language pairs; it is also a procedure that is widely used by many commercial language service providers. In previous work with the Spanish-English language pair, we found our approach feasible even among lay users with minimal training; these conditions closely mirror the public health context, where resources for training and calibration are limited.

There are several potential reasons for the preference for the HT over the MT+PE in this study:

#### Differences in MT Quality

Chinese machine translations have a different relative frequency of certain error types and lower quality overall. Compared to our previous studies on English-Spanish [8,13], we found that the Chinese translations had high percentages of word order and word sense errors, which require more cognitive effort to correct [14-16]. Adequacy and fluency also had lower ratings compared to the Spanish translations: adequacy for Chinese was 3.3 compared to 4.2 for Spanish; fluency was 3.1 versus 3.7 for Spanish. It should be noted that these scores are not directly comparable since the the sets of English documents used in these two studies were not identical; however, the differences in scores confirm the common observation in the MT community that MT for English-Chinese is less effective than for English-Spanish.

#### Instructions Provided to Posteditors

Posteditors might have misinterpreted the postediting instructions. Specifically, the instruction to “postedit only where necessary” and to not “rewrite” might have led them to produce fewer edits than they would under real-life circumstances. Quality raters observed that the postedited documents often contained very literal word-by-word translations that were perceived as unacceptable. In other language pairs with similar linguistic structures (like English and Spanish), more literal translations may still yield acceptable translation outputs, whereas fluent Chinese requires the translator to depart more strongly from a literal translation. Due to time and resource constraints for this study, as with prior studies, there also was no extensive training and calibration phase for the study participants. Combined with the lower quality of initial MT Chinese versions, the postediting instructions might help explain the lesser quality of the postedited Chinese translations as compared to the Spanish translations.

#### Linguistic Expertise of Posteditors

Although posteditors were selected for bilingual competence and familiarity with the domain of public health, they did not have to undergo initial language or translation tests to verify their editing abilities.

#### Engagement of Posteditors

Posteditors may not have been sufficiently engaged in the task, or they may have optimized for time rather than quality.

#### Different Levels of Quality Control

In the postediting, only one round of postediting was performed, followed by the quality rating task. We do not know how many iterations of editing and quality control were applied to the human-generated translations, since they were collected from different sources where the translation processes were not transparent. Our prior investigations into health department translation processes revealed that most of the public health HT documents had been translated in-house or by language service providers who conduct several rounds of postediting and review prior to making them public [7].

### Additional Follow-Up

In order to ascertain the contribution of these factors to the overall results, we conducted additional follow-up studies investigating the role of posteditor expertise, instruction, and engagement.

#### Expertise

To assess whether posteditor expertise played a role in the translation quality, we engaged the services of a public health professional who performed translation for a large metropolitan health department in Washington State (P6). She was given the original set of instructions to correct only as much as needed and to not rewrite the text extensively. She postedited four documents, which were then given as a set and blindly rated against their original human translations by five native Chinese speakers so that each rater reviewed all four documents. Three of the 5 raters selected the human translation over the MT+PE for all four documents; 2 raters rated one of the HT and MT+PE documents as equivalent.

#### Instructions and Engagement

To test whether our instructions to postedit only where necessary played a role in the MT+PE ratings, we modified the instructions to emphasize quality and recruited 3 posteditors to come back for another postediting session with the new instructions. The original instructions—as adapted from the Spanish study—directed posteditors to not alter a correct translation, even if it was not their first choice; to not engage in extensive rewriting of the text; and to not spend an extended period of time looking up grammar, punctuation, or unfamiliar terminology online. The updated instructions directed posteditors to use as much time and effort as necessary to ensure a high-quality translation. The 3 returning posteditors corrected a total of 10 documents, which were then blindly rated by a quality rater with language and public health expertise. As anticipated, posteditors took longer to produce the MT+PE translations with the updated instructions: P2’s average speed dropped from 35.38 CPM to 23.43 CPM, P4’s fell from 54.33 to 17.46 CPM, and P5’s decreased from 53.96 to 19.69 CPM. The rater chose the manual human translations for 6/10 documents, while rating four as equivalent—a notable improvement over the original instructions.

## Discussion

### Principal Findings

Although our prior research on English to Spanish translation indicated that MT+PE could produce translations equivalent in quality for less time and cost, our current study on the English-Chinese language pair showed that maintaining quality through postediting was more problematic. Translation between English and Chinese presents a challenge due to very divergent syntactic structures (eg, topic-comment structure in Chinese vs subject-verb-object structure in English), frequent dropping of pronouns in Chinese, higher degree of morphology in English, and other linguistic differences. Compared to a language pair like English and Spanish, SMT for English and Chinese generally tends to produce lower-quality results (eg, the results obtained in benchmark evaluations for different language pairs conducted by the US National Institute of Standards and Technology [17].

### Strengths and Limitations

Although, theoretically, professional translators with sufficient training and time should be able to produce an equivalent product through postediting MTs, even with instructions to take the time to provide the best quality translation, the final postedited translations still contained obvious errors that led the quality raters to prefer HTs in most cases. Experienced translators who performed the translations rated the adequacy and fluency of MT+PE lower in general than their less experienced counterparts and commented that for many machine-translated sentences it would be easier to start with the English version than to correct the MT version. However, it should be noted that our prior evaluation of health department translation processes found that HT documents undergo multiple editing cycles to ensure translation quality and cultural appropriateness. In the studies reported on here, the machine-translated documents underwent only one round of postediting. It is likely that with additional rounds of editing the MT+PE product would be further improved.

Another possible limitation of our study is the use of a single translation engine, Google Translate. However, most SMT systems are based on the same set of underlying statistical models, suggesting that the types and relative frequencies of translation errors would not have been significantly different had a different SMT system been used.

Additional work is needed to improve the quality of MT from English to Chinese. Word sense and word order errors require the most attention for improvement. Our team is currently working to improve these errors. In addition, particular care must be taken in selecting posteditors, documents, and machine translation engines, and in designing postediting instructions and quality control processes.

### Conclusion

In the United States, Chinese is the second most common language spoken by LEP individuals and the single most common character language used. However, due to the resources and time involved in human translation, health departments currently offer few health promotion materials in Chinese. Our investigation into the use of MT+PE to produce translations indicates that using the methods that worked for English to Spanish translations was not as effective with translation from English to Chinese. Multiple factors, including quality of MT and expertise of posteditors, may have contributed to these results. Our preliminary follow-up studies suggest that reducing word sense errors and word order errors would improve English to Chinese MTs, while additional training and expertise of bilingual posteditors may be needed in order to successfully apply online MT technology to public health practice. We are performing additional studies to determine how best to improve translation from English to Chinese in order to ensure quality translation at a low cost.

## Appendix

Multimedia Appendix 1 Study source documents and postediting times.

## Abbreviations

CPM: characters per minute
HT: human translation
LEP: limited English proficiency
MT: machine translation
NIH: National Institutes of Health
PE: postediting
SMT: statistical machine translation
