# Supplementary material for: Machine Translation of Public Health Materials From English to Chinese: A Feasibility Study
Source: JMIR Public Health Surveill. 2015 Nov 17;1(2):e17. doi: 10.2196/publichealth.4779 (PMC4869219; doi:10.2196/publichealth.4779)
Supplement: Multimedia Appendix 1 [file publichealth_v1i2e17_app1.pdf]

Study source documents and post-editing times

**Source Agency**	**Topic**	**Topic**	**Document type**	**Characters**	**Avg. CPM**
PHSKC	Vaccines	Hypothermia	Informational website	609	24.3
CDC	SARS	Vaccines	Informational website	1449	42.3
PHSKC	Acute Radiation Syndrome	Ringworm	Agency Letter	734	36.9
PHSKC	Chickenpox	Pink eye	Agency Letter	872	28.6
PHSKC	Cold sores	Cold sores	Agency Letter	756	33.9
PHSKC	Hand, foot & mouth disease				

Hand, foot & mouth disease
Agency Letter
773
35.5
PHSKC
Pink eye
Chickenpox
Fact Sheet
749
27.4
CDC
Ringworm
Acute Radiation Syndrome
Fact Sheet
738
49.9
CDC
Hypothermia
SARS
Fact Sheet
1294
79.6
WADOH
Sewage spill
Sewage spill
Fact Sheet
703
30.0
WADOH
Earthquakes
Earthquakes
Fact Sheet
782
29.9
WADOH
Floods
Floods
Fact Sheet
803

21.2

WADOH

Calling 911

Calling 911

Fact Sheet

754

36.4

WADOH

Pneumonic plague

Pneumonic plague

Fact Sheet

628

39.8

WADOH

Viral Hemorrhagic Fevers

Viral Hemorrhagic Fevers

Fact Sheet

1039

67.5

WADOH

Vitamin D for your child

Vitamin D for your child

Fact Sheet

1015

57.2

WADOH

Hypothermia

Hypothermia

Fact Sheet

607

18.5

MNDOH

Basic food safety

Basic food safety

Fact Sheet

568

21.0

MNDOH

Disaster clean-up tips

Disaster clean-up tips
Fact Sheet
609
24.0
DPHLA
Carbon monoxide poisoning
HIV facts
Fact Sheet
597
26.2
DPHLA
HIV facts
High blood pressure
Fact Sheet
568
33.4
DPHLA
High blood pressure
Exercise
Fact Sheet
499
39.9
PHSKC
Exercise
Strep Throat
Fact Sheet
856
67.0
NYCDOH
Strep Throat
Carbon monoxide poisoning
Informational Brochure
622
47.1
